# Ionic, not the osmotic component, is responsible for the salinity-induced inhibition of greening in etiolated wheat (*Triticum aestivum* L. cv. Mv Béres) leaves: a comparative study

**DOI:** 10.1007/s00425-023-04255-4

**Published:** 2023-10-20

**Authors:** Adél Sóti, Roumaissa Ounoki, Annamária Kósa, Beata Mysliwa-Kurdziel, Éva Sárvári, Katalin Solymosi

**Affiliations:** 1 Department of Plant Anatomy, Institute of Biology, Faculty of Science, ELTE Eötvös Loránd University, Budapest, Hungary; 2 Department of Plant Physiology and Biochemistry, Faculty of Biochemistry, Biophysics and Biotechnology, Jagiellonian University, Kraków, Poland; 3 Department of Plant Physiology and Molecular Plant Biology, Institute of Biology, Faculty of Science, ELTE Eötvös Loránd University, Budapest, Hungary

**Keywords:** Chloroplast, Etioplast, Greening, Osmotic stress, Prolamellar body, Salt stress

## Abstract

**Main conclusion:**

Greening was partially (in 300 mM NaCl, CaCl_2_, 600 mM KNO_3_ or KCl) or fully inhibited (in 600 mM NaCl, NaNO_3_ or NaCl:KCl) by the ionic and not the osmotic component of salinity.

**Abstract:**

Although high soil salinity is an increasing global problem, not much is known about how direct exposure to salinity affects etiolated leaves of seedlings germinating in the soil and then reaching the surface. We investigated the effect of various salt treatments on the greening process of leaves in 8- to 11-day-old etiolated wheat (*Triticum aestivum* L. Mv. Béres) seedlings. Etiolated leaf segments pre-treated on different salt (600 mM NaCl:KCl 1:1, 600 mM NaCl, 600 mM KCl, 600 mM NaNO_3_, 600 mM KNO_3_, 300 mM KCl, 300 mM NaCl or 300 mM CaCl_2_) or isosmotic polyethylene glycol 6000 (PEG) solutions for 1.5 h in the dark and then greened for 16 h on the same solutions were studied. Leaf segments greened on PEG (osmotic stress) or on 300 mM KCl had similar chloroplasts compared to control samples greened on Hoagland solution. Slightly slower development of chloroplast structure and function (photosynthetic activity) was observed in segments greened on 300 mM NaCl or CaCl_2_, 600 mM KNO_3_ or KCl. However, etioplast-to-chloroplast transformation and chlorophyll accumulation were fully inhibited and peculiar prothylakoid swelling occurred in segments greened on 600 mM NaCl, NaNO_3_ or NaCl:KCl (1:1) solutions. The data indicate that not the high osmolarity of the used salt solution, but its ions, especially Na^+^, had the strongest negative impact on these processes.

**Graphical abstract:**

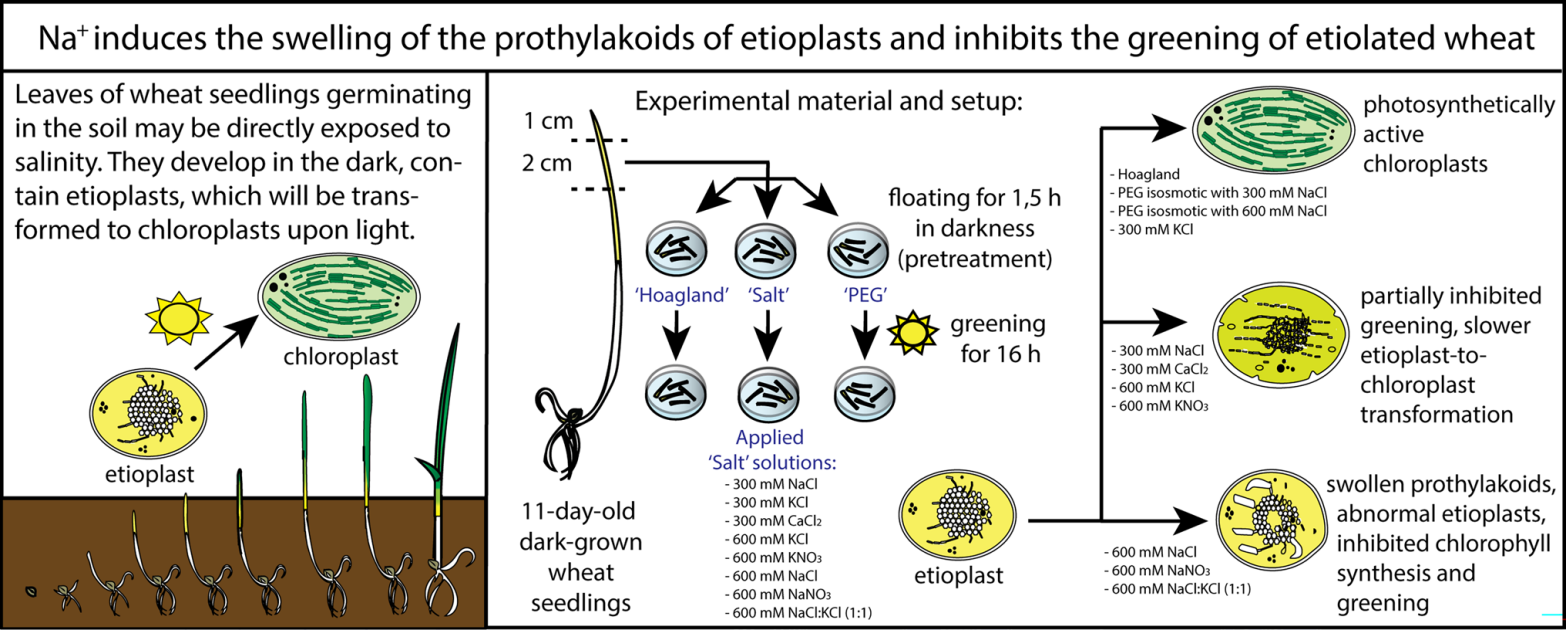

**Supplementary Information:**

The online version contains supplementary material available at 10.1007/s00425-023-04255-4.

## Introduction

The high salinity of the soil is a very serious and global problem in agriculture. According to estimations, it affects about 5–7% of the world’s arable land (about 77 million hectares) (Maathuis et al. 2014), and due to improper land use and irrigation practices, this area is constantly increasing. Because of the varying quality and quantity of precipitation and infiltrating ground or seawater, as well as the evapotranspiration, the salinity and the sodicity of the soils are constantly changing (El Sabagh et al. 2021). Therefore, salt-affected sites have a large variation in surface salinity and available soil water depending on external temperature and other factors; thus, salt concentrations in soil water are fluctuating over time.

In general, but especially in glycophytes (plants that cannot tolerate ≥ 200 mM NaCl in the soil (İbrahimova et al. 2021), salt stress negatively influences seed germination and seedling establishment (Malcolm et al. 2003), and has a serious effect on plant growth and yield even at later stages of development (El Sabagh et al. 2021). The presence of different ions at high concentration in the soil inhibits the water uptake of plants and causes hyperosmotic stress, whilst ionic effects are also induced if ions enter the plants’ roots via various uptake mechanisms (Isayenkov and Maathuis 2019). In addition, salt ions (Na^+^, Cl^−^) negatively affect the uptake of other essential ions and thus disturb the ion homeostasis of the plants (Isayenkov and Maathuis 2019). The accumulation of excess Na^+^ and Cl^−^ within the cells may result in an osmotic imbalance, but the ions may also have direct toxic effects on plant metabolism (e.g. by causing oxidative stress) (Isayenkov and Maathuis 2019; İbrahimova et al. 2021). Due to the above described complex negative effects caused by salt stress, the photosynthetic activity of crops is often decreased resulting in yield loss.

Plant photosynthesis takes place in the green plastids, the chloroplasts. In seedlings developing under natural light conditions, chloroplasts differentiate directly from proplastids (Yadav et al. 2019). However, in the absence of light, chlorophyll (Chl) biosynthesis and the synthesis of Chl–protein complexes are inhibited in angiosperms, and proplastids turn into etioplasts (Armarego-Marriott et al. 2020). The inner membranes of etioplasts are organised into a highly regular tubuloreticular membrane system termed prolamellar body (PLB) interconnected with flat prothylakoid lamellae (Solymosi and Aronsson 2013; Armarego-Marriott et al. 2020). Upon illumination, the PLBs are quickly rearranged into the mature thylakoid system of chloroplasts (Kowalewska et al. 2016; Armarego-Marriott et al. 2020) and active synthesis of photosynthetic pigments and thylakoid components occur (Solymosi and Aronsson 2013; Kowalewska et al. 2016). Light is essential for the transformation of protochlorophyllide (Pchlide) to chlorophyllide (Chlide) (Solymosi and Mysliwa-Kurdziel 2021). Pchlide molecules located in the active centre of the NADPH:Pchlide oxidoreductase enzyme (LPOR—E.C. 1.3.1.33) are transformed directly into Chlide, and thus their fluorescence emission at 655 nm disappears and the fluorescence of Chlide appears at 690 nm. (The fluorescence emission maxima referred to in our text all indicate values measured at 77 K temperature.) If the leaves are kept at room temperature after illumination, the maximum is gradually shifted towards 680 nm during the so-called Shibata shift (Smeller et al. 2003). Within 2–4 h after illumination, the Chl molecules get incorporated to Chl–protein complexes with fluorescence emission maximum at 684 nm (Franck et al. 1997). Finally, in parallel with the appearance of the fully active photosynthetic apparatus, three dominant bands appear: two of them having maxima at 685 and 695 nm originate from the core antennas of photosystem II (PSII), and the third one around 735 nm originates from the light-harvesting complexes of photosystem I (PSI) (Kalaji et al. 2017; Lamb et al. 2018).

Most works dealing with the various effects of salt stress on plastid structure and function have been carried out on chloroplasts of fully developed, light-grown plants. Nevertheless, only a few data are available on salt stress in developing plants (Abdelkader et al. 2007a, b; Srivastava and Sharma 2021) or in other plastid types, for instance, etioplasts typical for soil-germinated agricultural crops (Kakuszi et al. 2016), which may indeed be directly exposed to soil salinity. Salt stress was shown to cause the swelling of the lumen of the prothylakoids in etioplasts, to inhibit Chl biosynthesis and the etioplast-to-chloroplast transformation (Abdelkader et al. 2007a, b). However, these experiments were performed using 600 mM NaCl:KCl (1:1) solution, which is considered a relatively high concentration (equal to sea salinity level), relevant naturally only in extreme environments with very saline ground water, in areas with seawater seepage or in case of tsunamis. Therefore, the questions can be raised, (i) whether the observed physiological and structural alterations are caused by the osmotic or ionic components of salt stress. Also, (ii) which salt stress-related ions and at which concentration these are responsible for the alterations. To this aim, we compared the effect of iso-osmotic polyethylene glycol 6000 (PEG) treatments with various salt stress treatments on the etioplast-to-chloroplast transformation in etiolated wheat leaves.

## Materials and methods

### Plant material and growth conditions

The wheat seedlings (*Triticum aestivum* L. cv. Mv Béres, Agricultural Institute, Martonvásár, Hungary) were germinated and grown hydroponically in tap water for 8–11 days in the dark. For the treatments, we discarded the 1 cm long leaf tip region and used the next 2 cm long leaf segments of the plants. Leaf pieces were treated in parallel with different solutions: Hoagland solution as control (osmolarity 8–9 mOsm, referred to as’Hoagland’), PEG 6000 dissolved in Hoagland solution with a final osmolarity of 1050 mOsm (referred to as 600 PEG henceforth) and 579 mOsm (referred to as 300 PEG later), corresponding to the osmolarity of 600 mM and 300 mM NaCl solutions, respectively, as well as with different salt solutions dissolved in Hoagland (600 mM 1:1 NaCl:KCl, 300 mM NaCl, 600 mM NaCl, 300 mM KCl, 600 mM KCl, 300 mM CaCl_2_, 600 mM KNO_3_, and 600 mM NaNO_3_). PEG was purchased from Hungary Biocenter Ltd. Unless otherwise stated, other chemicals were bought from Reanal Ltd. (Budapest, Hungary) or VWR Ltd. (Radnor, PA, USA). The solutions were always freshly prepared, and 4–10 pieces of 2-cm leaf segments were placed in 15 ml of the solution, usually in a small Petri dish. To obtain higher amount of material for Blue Native polyacrylamide gel electrophoresis (BN PAGE), several (100 or more) leaf segments were floated in large Petri dishes taking care that leaf segments do not cover each other. All manipulations with etiolated material were performed under green light which does not induce the transformation of Pchlide to Chlide. The etiolated leaf segments were floated in treatment solutions for 1.5 h in the dark as pretreatment, and after that, they were greened for 16 h under continuous white light (50 μmol photons m^−2^ s^−1^ photon flux density) at room temperature. It should be noted, however, that some uncertainties were observed amongst individual plants’ greening abilities even within the same experiment. Therefore, our data represent the conclusive general results of at least 3–6 repetitions of the treatments (the number of biological replicates, n, is provided for each measurement separately).

### Determination of chlorophyll and carotenoid content

Pigments were extracted with 80% (v/v) acetone buffered in 5 mM N-(tri(hydroxymethyl)methyl)glycine (Tricine) KOH pH 7.8, and the absorption spectra of the centrifuged extracts were measured spectrophotometrically by a UV–VIS spectrophotometer (UV-1601, Shimadzu, Kyoto, Japan). Pigment contents were determined using the equations of Lichtenthaler (1987) for carotenoids and Porra et al. (1989) for chlorophylls, respectively.

### Fluorescence spectroscopy

The 2 cm long leaf segments were placed in glass tubes with an internal diameter of 3 mm and then frozen in liquid nitrogen. The leaf pieces were fixed in the tube with a drop of water. The samples were kept in liquid nitrogen throughout the measurements. Acquisition of the 77 K fluorescence emission spectra was achieved using a Jobin Yvon Horiba Fluoromax-3 (Jobin Yvon Horiba, Longjumeau, France) spectrofluorimeter. Data were collected every 0.5 nm in the 580–780 nm range with an integration time of 0.1 s. The excitation and the emission slits were set to 2 and 5 nm, respectively. Emission spectra of leaf segments were recorded using excitation at 440 nm. During the measurement of each leaf piece, spectra were recorded three times in each case, and their average was calculated automatically and used for further studies.

During room temperature fluorescence analyses of acetonic pigment extracts of the samples, we used 430 nm and 460 nm excitations, to selectively excite Chl *a* and *b*, respectively.

Fluorescence emission spectra were analysed using SPSERV V3.14 software (copyright Csaba Bagyinka, Institute of Biophysics, Hungarian Academy of Sciences). First, 3- and 5-point smoothings were performed to remove various noises arising from electrical equipment, light scattering, or the movement of bubbles in liquid nitrogen. As the sensitivity of the fluorimeter's detector depends on the wavelength, the raw spectra had to be multiplied with a correction curve in order to compensate for this. Finally, if necessary, a baseline correction was performed by subtracting an appropriate Gaussian curve from the spectrum. Further spectral analyses and averaging were performed using the corrected spectra in Excel. Spectra were normalised to their maxima and then averaged for each treatment. These normalised and averaged spectra are shown and compared in the figures.

### Photosynthetic activity

The photosynthetic parameters were measured based on in vivo chlorophyll fluorescence using FluorPen FP 100 (Photon Systems Instruments, Drásov, Czech Republic) handheld device as described earlier (Ounoki et al. 2021). Chl fluorescence induction kinetics were recorded in light- and in 20-min dark-adapted samples (to obtain “Qy light”, equivalent to *F*_v_′/*F*_m_′ or “Qy dark”, equivalent to *F*_v_/*F*_m_ values). As light-adapted samples, we used leaf segments that have been constantly illuminated by 50 μmol photons m^−2^ s^−1^ photon flux density during greening. These parameters describe the photochemical activity and structural dynamics of PSII (Björkman and Demmig 1987). Chl fluorescence quantum yield was induced by saturating light (2050 µmol photons m^−2^ s^−1^ photon flux density) applied for 1 s in case of Qy measurements and for 2 s in case of the OJIP fluorescence transient measurements (Strasser and Govindjee 1992; Strasser et al. 2000) using the above instrument.

The following parameters were automatically calculated by the instrument from the recorded OJIP curves according to the following formula as described in the instrument’s instruction manual:

*F*_O_ = *F*_50μs_, fluorescence intensity at 50 μs.

*F*_J_ = fluorescence intensity at J-step (at 2 ms).

*F*_I_ = fluorescence intensity at I-step (at 30 ms).

*F*_m_ = maximal fluorescence intensity.

*F*_v_ = *F*_m_−*F*_O_ (maximal variable fluorescence).

*V*_J_ = (*F*_J_−*F*_O_)/(*F*_m_−*F*_O_) (the relative variable fluorescence at 2 ms).

*V*_I_ = (*F*_I_−*F*_O_)/(*F*_m_−*F*_O_) (the relative variable fluorescence at 30 ms).

Phi_Pav = time to reach *F*_m_ (in ms).

### Transmission electron microscopy (TEM)

Middle regions of the treated 2-cm long leaf segments were dissected into 1 mm wide stripes in 2.5% (v/v) glutaraldehyde (buffered in 70 mM Na_2_HPO_4_–KH_2_PO_4_, pH 7.2) and kept for at least 3 h after sampling in the same fixative, before being post-fixed in 1% OsO_4_ (w/v) in the same buffer for 2 h. Samples were washed with the buffer three times for 15 min after each fixation step. After fixation, samples were dehydrated in ascending ethanol series. Samples were then embedded in Durcupan ACM resin (Fluka, Buchs, Switzerland) using propylene-oxide as intermediate solvent. All fixation and embedding steps were carried out at room temperature, the resin was polymerised for 72 h at 62 °C. Reichert Jung ultramicrotome (Reichert Jung AG, Vienna, Austria) equipped with a diamond knife was used for ultrathin (70 nm) sectioning. Sections transferred to 300 mesh copper grids were then stained with uranyl acetate and Reynold’s lead citrate. TEM investigations were performed with a JEOL JEM 1011 (JEOL Ltd., Tokyo, Japan) at 80 kV accelerating voltage. Olympus Morada CCD camera was used to capture digital photographs (Olympus Optical Co. Ltd., Tokyo, Japan), and representative images were chosen for presentation.

### Isolation of thylakoid complexes

Solutions were removed from the leaf surface by quick washing with distilled water in a Nutch filter. For isolation of etio-chloroplasts, leaves were homogenised in isolating buffer (50 mM HEPES–KOH, pH 7.0, 330 mM sorbitol, 2 mM EDTA disodium salt, 2 mM MgCl_2_, 0.1% (w/v) bovine serum albumin, 0.1% (w/v) Na ascorbate) at 4 °C for 3 × 3 s by Waring Blender in dim light immediately after 16 h greening. After filtration through four layers of gauze and two layers of Miracloth™ (Calbiochem-Novabiochem, San Diego, CA, USA), the homogenate was immediately centrifuged in a swing-out rotor (3000 *g*, 5 min, 4 °C), and washed in isolating buffer without BSA and Na ascorbate. Isolation of thylakoid membranes was carried out using osmotic shock of plastids by 10 mM sodium pyrophosphate (5000 g, 5 min, 4 °C). To remove large part of CF_1_, washing with 5 mM Tricine-(CH_3_)_4_NOH (pH 7.5), 0.1 M sorbitol was used (Fuad et al. 1983). After starch removal (100 g, 3 min, 4 °C), the membranes were pelleted from the supernatant with 10,000 g, 10 min, 4 °C. Thylakoids were stored in liquid nitrogen dissolved in Tris(hydroxymethyl)aminomethane (Tris)-maleate (pH 7.0), 35% (w/v) glycerol.

To separate thylakoid complexes and to determine their polypeptide patterns, first-dimensional BN PAGE and second-dimensional denaturing SDS-PAGE were performed, respectively (Sárvári et al. 2022). For BN PAGE, thylakoids (250 µg Chl ml^−1^; µg protein µg^−1^ Chl ~ 10) were solubilised using 2% *n*-dodecyl-*β*-d-maltoside plus 1% digitonin and separated in 4.5–12% BN gel gradient. SDS-PAGE of the about 3 mm wide lanes cut out of BN gels was carried out in 10–18% gradient gels (Laemmli 1970). The identity of the complexes was recognised as in Basa et al. (2014).

### Statistical analyses

GraphPad Prism 8 was used to perform statistical analyses (normality test, ANOVA, and post hoc tests). Tukey–Kramer multiple comparisons test was used as a post hoc test when data had a normal distribution and significant differences were found by 1-way ANOVA. In case of data not following normal distribution, the Kruskal–Wallis non-parametric ANOVA test was used, followed by the Dunn test as a post hoc test. Different letters were assigned to significant differences. *P* < 0.05 was considered significant for all data.

## Results

### Leaf phenotype and morphology

The leaf segments treated with high osmolarity (600 PEG, and 600 mM solutions) were strongly rolled, whilst the leaf blades were fully flattened in the Hoagland sample. In case of samples with medium osmolarity (300 PEG and 300 mM solutions), the leaves had an intermediate curled phenotype. This indicates that except for Hoagland, the different solutions cause different extent of osmotic stress to the leaves.

After 16 h of illumination, colour differences amongst the various applied treatments were clearly visible: leaf segments were the greenest when floated on Hoagland solution, became gradually lighter green when floated on PEG and medium concentration (300 mM) salt solutions, and remained yellow in treatments with high (600 mM) salt concentrations (not shown). The photosynthetic pigments were extracted in order to quantify the observed differences in greening.

### Photosynthetic pigment contents

When compared with the control, i.e. leaf segments floating on Hoagland solution, the Chl content was reduced by all types of treatment. The order of the induced changes was the following: Hoagland < 300 PEG < 600 PEG, 300 mM KCl, 300 mM NaCl < 300 mM CaCl_2_ < 600 mM KNO_3_, 600 mM KCl, 600 mM NaNO_3_, 600 mM NaCl, 600 mM NaCl:KCl (1:1) (Table 1). At 600 mM salt concentration, no significant difference was found amongst the pigment contents of the leaves greened on the used solutions. In general, the Chl *a/b* and the carotenoid/Chl ratio of leaves increased in parallel with the decrease in the Chl content. The Chl *a/b* ratios were > 5 in 600 mM salt treatments expect for 600 mM NaNO_3_. Due to the very low Chl content of the samples, we also performed fluorometric determinations, which revealed only low amounts or traces of Chl *b* in these samples (not shown).

**Table 1:** Average pigment compositions with standard error values of etiolated wheat (*Triticum aestivum* L. cv. Mv Béres) leaf segments floated on various solutions for 1.5 h in the dark and then greened for 16  h on the same solution

	Chl *a* + *b* μg g^−1^ FM	Chl *a*/*b*	Car μg g^−1^ FM	Car/Chl
Hoagland	515.90 ± 21.60^a^	3.95 ± 0.06^a^	173.00 ± 5.49^a^	0.35 ± 0.01^a^
300 PEG	422.10 ± 46.82^ab^	4.17 ± 0.08^ad^	168.40 ± 12.21^ab^	0.41 ± 0.03^ab^
600 PEG	310.60 ± 22.22^abc^	4.28 ± 0.18^acd^	126.50 ± 8.13^abcd^	0.43 ± 0.01^abd^
300 mM KCl	327.50 ± 29.05^ab^	4.43 ± 0.15^abe^	160.90 ± 6.01^abd^	0.51 ± 0.03^abc^
300 mM NaCl	302.00 ± 15.99^ab^	4.44 ± 0.14^abe^	153.90 ± 10.01^abde^	0.48 ± 0.03^ae^
300 mM CaCl_2_	99.83 ± 22.34^bc^	5.06 ± 0.35^abe^	115.10 ± 6.47^bf^	1.74 ± 0.44^bef^
600 mM KNO_3_	27.26 ± 5.27^bc^	8.15 ± 0.59^be^	105.30 ± 7.95^bf^	4.77 ± 1.01^cde^
600 mM KCl	25.32 ± 5.14^ cd^	5.48 ± 0.74^ae^	96.51 ± 3.93^cf^	4.97 ± 0.85^cde^
600 mM NaNO_3_	13.13 ± 0.77^c^	3.99 ± 0.73^ad^	96.03 ± 6.80^cef^	6.90 ± 0.31^ce^
600 mM NaCl	16.63 ± 1.35^ cd^	6.94 ± 0.62^ce^	106.90 ± 3.09^bf^	6.53 ± 0.63^cfg^
600 mM NaCl:KCl (1:1)	19.86 ± 3.84^ cd^	6.06 ± 0.46^de^	102.20 ± 8.32^df^	4.15 ± 0.55^beg^

Applied solutions: Hoagland, 600 PEG, 300 PEG, 300 mM NaCl, 300 mM KCl, 300 mM CaCl_2_, 600 mM KNO_3_, 600 mM NaNO_3_, 600 mM KCl, 600 mM NaCl, and 600 mM NaCl:KCl (1:1). FM—fresh mass, Chl—chlorophyll, Car—carotenoids. Car/Chl is the ratio of the total Car values and the total Chl *a* + *b* values for each sample, used to estimate the greening stage of the sample. Mean values with standard errors are provided. Different letters indicate statistically significant differences between the samples according to Kruskal–Wallis non-parametric ANOVA followed by Dunn’s multiple comparisons test (*P* < 0.05) (*n* = 3–19)

### Spectral characteristics of leaves

**Fig. 1:**
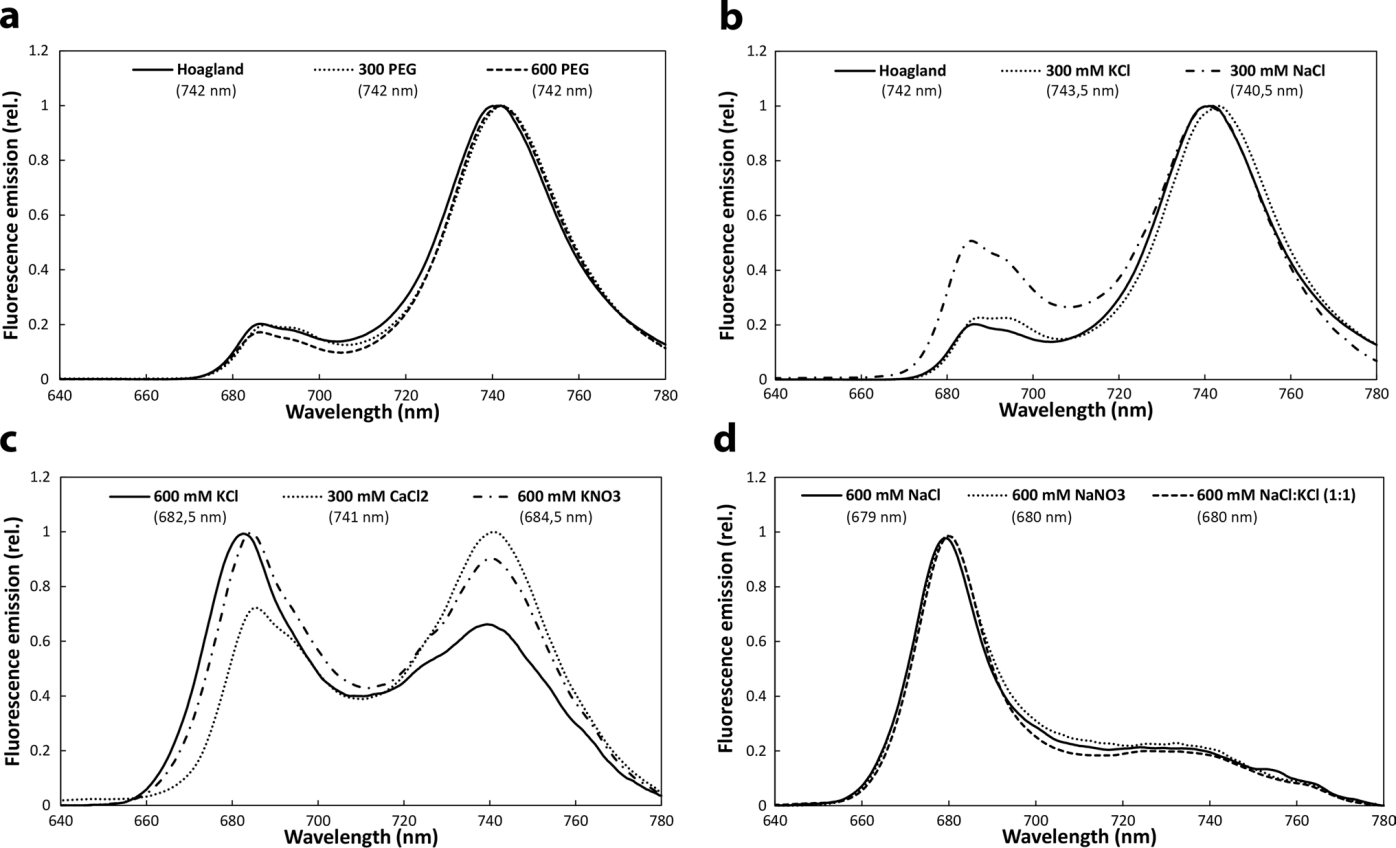
Normalised and averaged 77 K fluorescence emission spectra of etiolated wheat (*Triticum aestivum* L. cv. Mv. Béres) leaf segments floated on various solutions for 1.5 h in the dark and then greened for 16 h on the same solution. Applied solutions: Hoagland, 300 PEG, 600 PEG (**a**), Hoagland, 300 mM KCl, 300 mM NaCl (**b**), 300 mM CaCl_2_, 600 mM KCl, 600 mM KNO_3_ (**c**), 600 mM NaCl, 600 mM NaNO_3_, 600 mM NaCl:KCl (1:1) (**d**). Excitation wavelength: 440 nm. The positions of the fluorescence emission maxima of the spectra are also indicated in parenthesis for all treatments (*n* = 11–44)

After 16 h of illumination, 77 K fluorescence emission spectra of leaf segments treated with 300 PEG and 600 PEG were similar to the control (Hoagland) and characteristic to green leaves showing maxima at around 685, 695, and 740 nm (Lamb et al. 2018) (Fig. 1a, Table S1). Whilst the spectra of leaves greened with 300 mM KCl were similar to those greened on Hoagland solution, the relative intensity of the short-wavelength bands was higher in samples treated with 300 mM NaCl (Fig. 1b) referring to less well-organised photosystems. The 300 mM CaCl_2_-treated leaf segments had much lower Chl content than the above-mentioned ones, their spectra differed from those treated with 300 mM NaCl (compare Fig. 1b and c). The leaves treated with 600 mM KCl and 600 mM KNO_3_ also showed the fluorescence bands characteristic for green leaves, but with increased fluorescence in the short-wavelength region, and a small shoulder at 720 nm (Fig. 1c). The long-to-short-wavelength fluorescence emission ratio of the spectra of KNO_3_-treated samples was higher than in those treated with KCl, i.e. the nitrate anion had a more favourable effect on thylakoid development. Summing up, the spectra of leaves treated with 600 mM KCl or KNO_3_, 300 mM NaCl or CaCl_2_ showed intermediate characteristics and partial greening (Fig. 1b and c), i.e. the fluorescence of the Chl–protein complexes characteristic for PSI and PSII appeared already, but their intensities differed from that observed in leaves greened on Hoagland solution.

In contrast, in samples treated with 600 mM NaCl:KCl (1:1), 600 mM NaCl, and 600 mM NaNO_3_ solutions (Fig. 1d), the leaf segments remained yellow and their fluorescence emission maximum could be observed at approx. 680 nm.

Quantitative comparison of the ratios of the short- and long-wavelength emission maxima of the spectra of the different samples (Table S1) indicated significant increase in the non-greening samples with low Chl contents.

As the Chl contents (Table 1) and recorded 77 K fluorescence emission spectra (Fig. 1, Table S1) indicated clear differences in the greening stage of samples treated with the different solutions, we also wanted to compare the organisation of Chl–protein complexes in the thylakoid membranes, and plastid ultrastructure in the leaves treated with different solutions.

### Organisation of Chl–protein complexes

**Fig. 2:**
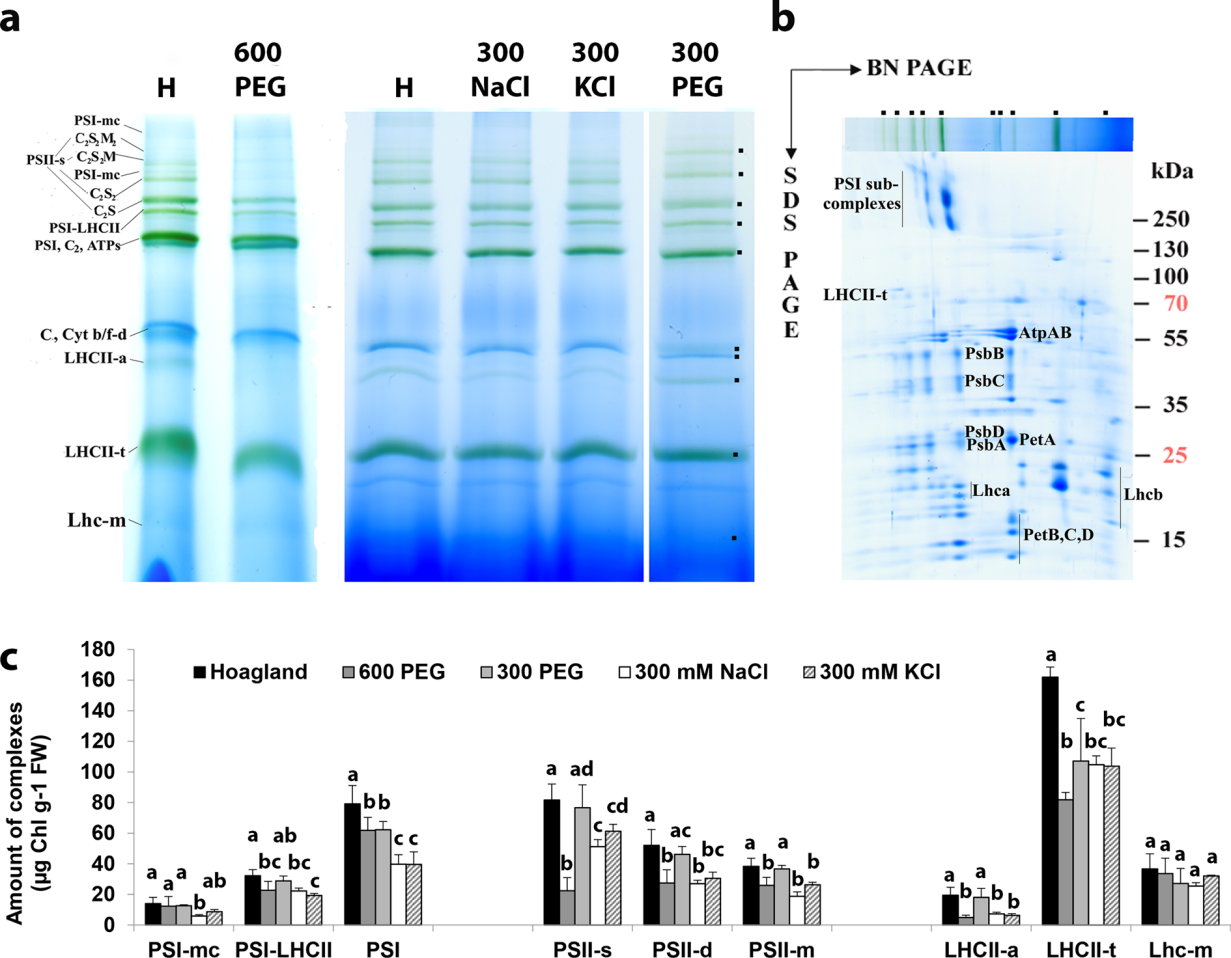
Thylakoid complexes present in the wheat (*Triticum aestivum* L. cv. Mv Béres) leaf segments floated on various solutions for 1.5 h in the dark and then greened for 16 h on the same solution. Applied solutions: Hoagland, 600 PEG, 300 PEG, 300 mM NaCl, 300 mM KCl. **a** Thylakoids (250 µg Chl ml^−1^; µg protein µg^−1^ Chl ~ 10) were solubilised using 2% n-dodecyl-*β*-D-maltoside plus 1% digitonin and separated in 4.5–12% BN gel gradient. PS, photosystem; LHC, Lhc—light-harvesting complex; LHCII-a—LHCII-assembly: CP29-CP24-LHCII trimer; mc, megacomplex; s, supercomplex; t, trimer; d, dimer; m, monomer; Cyt *b/f-d*, cytochrome *b*_*6*_*f* complex; ATPs, ATP synthase; C, core complex of PSII; S and M, strongly and moderately bound LHCII trimers, respectively. **b** 2D BN/SDS-PAGE of 300 PEG sample. Standard proteins: PageRuler™ Plus Prestained Protein Ladder (ThermoFisher Scientific 26,619, Lot #00803392). **c** Distribution of leaf Chl content amongst the complexes. Error bars represent SD values (*n* = 3–20). Different letters indicate statistically significant differences between the samples according to 1-way ANOVA followed by Tukey’s multiple comparisons test (*P* < 0.05)

The Chl–protein complexes in the thylakoid membranes were studied by BN PAGE. This method retains the native forms and interactions of the complexes (Schägger and von Jagow 1991; Kügler et al. 1997). The bands in BN PAGE patterns of wheat thylakoids (Fig. 2a) were identified as PSI, PSII, light-harvesting complexes (LHC, Lhc), cytochrome* b*_*6*_*/f* (Cyt *b/f*) and ATP synthase complexes in different assembly forms according to their polypeptide patterns (Fig. 2b) identified in Basa et al. (2014).

Greening of the etiolated wheat leaf segments was affected variously by the treatments. As it was mentioned earlier, 600 mM NaCl:KCl (1:1) treatment arrested greening totally, so thylakoid composition could be hardly studied. Mainly ATP synthase and Cyt *b/f* complexes were distinguishable in the isolated thylakoids (Fig. S1) like in pea etioplasts (Kanervo et al. 2008).

Amongst the PEG and 300 mM salt treatments, partial hindrance of greening was only induced by 600 PEG, where the accumulation of PSII, especially its oligomeric states (PSII-s) and LHCII were more retarded than that of PSI reaching 44%, 55%, and 77% of the control values obtained in samples greened on Hoagland solution, respectively (Fig. 2c). Compared to the Chl-containing complexes, the amount of the Cyt *b/f* complex was higher in the less developed 600 PEG thylakoids than in samples greened on Hoagland (Fig. S2). The effects of other treatments on thylakoid biogenesis were moderate (Fig. S2). The accumulation of PSI and PSII was more sensitive to 300 mM salts than to 300 PEG, whilst that of the light-harvesting complexes was similarly retarded by all these treatments. The accumulation of PSII was a little more negatively influenced by NaCl than KCl at 300 mM concentration.

**Fig. 3:**
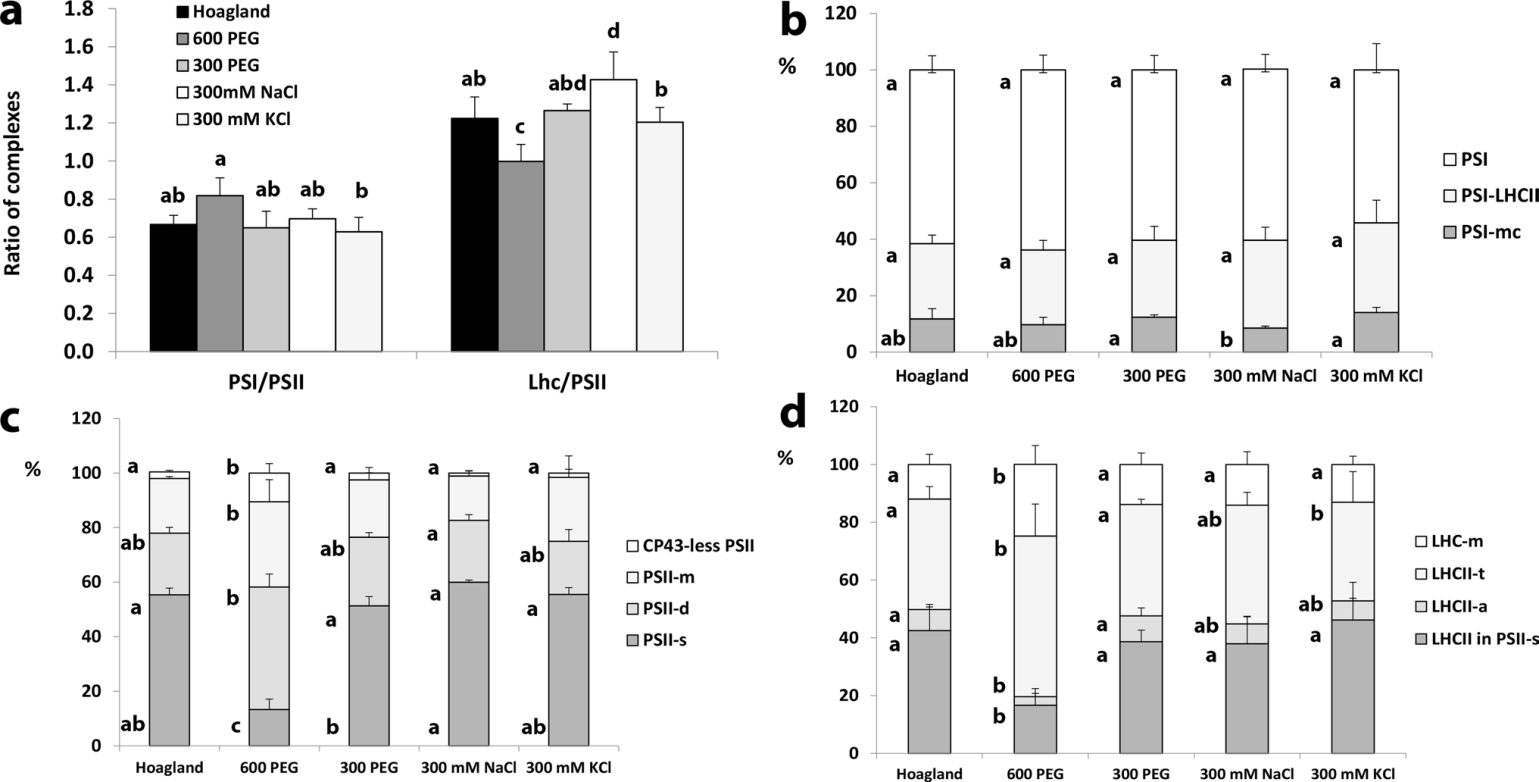
Ratios of thylakoid complexes and distribution amongst their assembly forms in the wheat (*Triticum aestivum* L. cv. Mv Béres) leaf segments floated on various solutions for 1.5 h in the dark and then greened for 16 h on the same solution. Applied solutions: Hoagland, 600 PEG, 300 PEG, 300 mM NaCl, 300 mM KCl. **a** Characteristic ratios of the main Chl-containing complexes calculated as the sum of PSI and PSII bands in the 1st-dimensional BN PAGE patterns, i.e. they contain the core complexes together with their antennae (Sárvári et al. 2022). Distribution of PSI (**b**), PSII (**c**), and LHCII complexes (**d**) amongst their different assembly forms calculated from the 1st-dimensional BN PAGE (PSI) and the 2nd-dimensional SDS-PAGE patterns (PSII, LHCII), respectively. Error bars represent SD values. Different letters indicate statistically significant differences between the samples according to 1-way ANOVA followed by Tukey’s multiple comparisons test or according to Kruskal–Wallis non-parametric ANOVA followed by Dunn’s multiple comparisons test (*P* < 0.05) (*n* = 4–14)

Regarding the general organisation of thylakoids, the PSI/PSII ratio (including core complexes and their antennae) only increased by the 600 PEG treatment, whilst the LHCII/PSII ratio was a little lower and higher in 600 PEG and 300 mM NaCl compared to the Hoagland treatment, respectively (Fig. 3a). The distribution of complexes amongst their different assembly forms was similar in the different treatments except for 600 PEG (Fig. 3b–d). We could not see any influence of the treatments on the PSI forms. However, this methodology was not optimised to retain megacomplexes, so we could not determine the amounts of PSI–PSII megacomplexes (Järvi et al. 2011). We only suppose that the amount of PSI-LHCII—very sensitive to solubilisation with n-dodecyl-*β*-D-maltoside even in the presence of 1% digitonin (Sárvári et al. 2022)—was proportional to its original amount. At the same time, the PSII supercomplex formation was retarded by 600 PEG treatment and the amount of CP43-less PSII was also the highest in 600 PEG treated leaves (see also Fig. S2).

### Photosynthetic activity

OJIP curves show the course of fast Chl-*a* fluorescence transients and can be used to characterise linear electron transport. The phases of the curve show the closing of PSII. In dark-adapted leaves, the O point represents the state where all reaction centres of PSII (P680) are ready to accept excitons (PSII open state). The O–J phase shows the reduction of Q_A_ to Q_A_^−^. The intermediate phase between J and I point characterises the filling of the plastoquinone pool (electron transport to PQ, PC, Cyt *b/f* etc.). The final point (P) shows the closed state of PSII where all the plastoquinone (PQ) molecules are reduced to PQH_2_ (Strasser and Govindjee 1992; Strasser and Srivastava 1995; Strasser et al. 2004; Küpper et al. 2019).

**Fig. 4:**
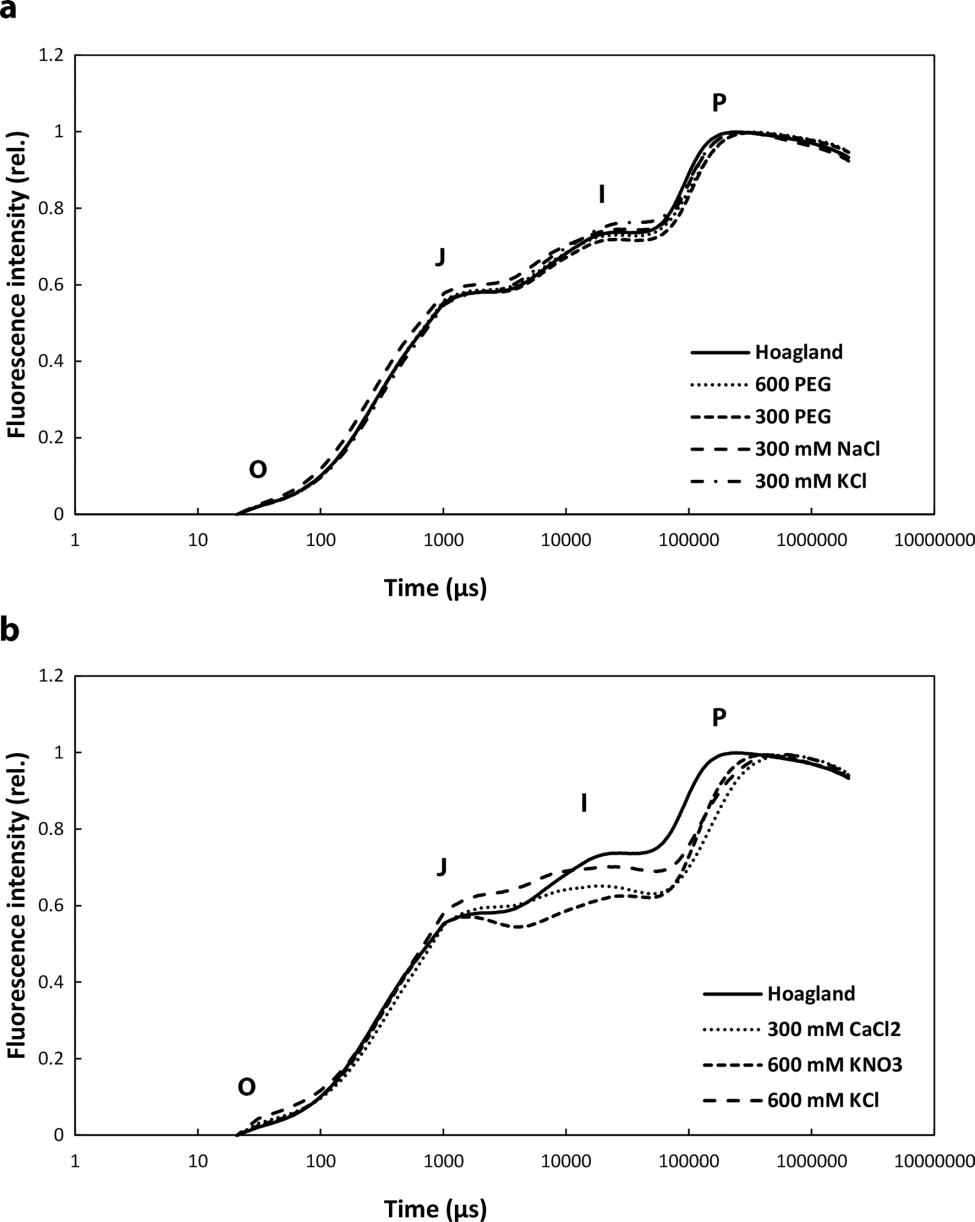
Fast chlorophyll a fluorescence OJIP transients of the wheat (*Triticum aestivum* L. cv. Mv Béres) leaf segments floated on various solutions for 1.5 h in the dark and then greened for 16 h on the same solution. Applied solutions: Hoagland, 600 PEG, 300 PEG, 300 mM NaCl, 300 mM KCl (**a**), Hoagland, 300 mM CaCl_2_, 600 mM KNO_3_, 600 mM KCl (**b**). Plants were dark-adapted for 20 min before the OJIP transients were recorded, and the transients were double normalised to F_0_ and F_m_ and then averaged (*n* = 10–17)

Greening of the leaf segments, and typical OJIP curves was observed in the following samples: Hoagland, 300 and 600 PEG, 300 mM NaCl, 300 mM KCl, 600 mM KCl, 600 mM KNO_3_, and 300 mM CaCl_2_ solutions (Fig. 4a, b). In samples which showed no accumulation of the photosynthetic Chl–protein complexes (Fig. 1d), no photosynthetic activity could be recorded (not shown). In the case of 300 and 600 PEG, as well as in samples treated with 300 mM NaCl and 300 mM KCl solutions, the course of the OJIP curve was the same as in the control (Hoagland) (Fig. 4a) but 300 mM CaCl_2_, 600 mM KCl, and KNO_3_ curves were modified a lot in the J-I-P fragment (Fig. 4b). In the plants treated with 600 mM KCl, the J point was higher than in all other treatments. In the case of 300 mM CaCl_2_ and 600 mM KNO_3_ treatments, the photochemical O–J phase is similar to that in the control (Hoagland) plants.

**Fig. 5:**
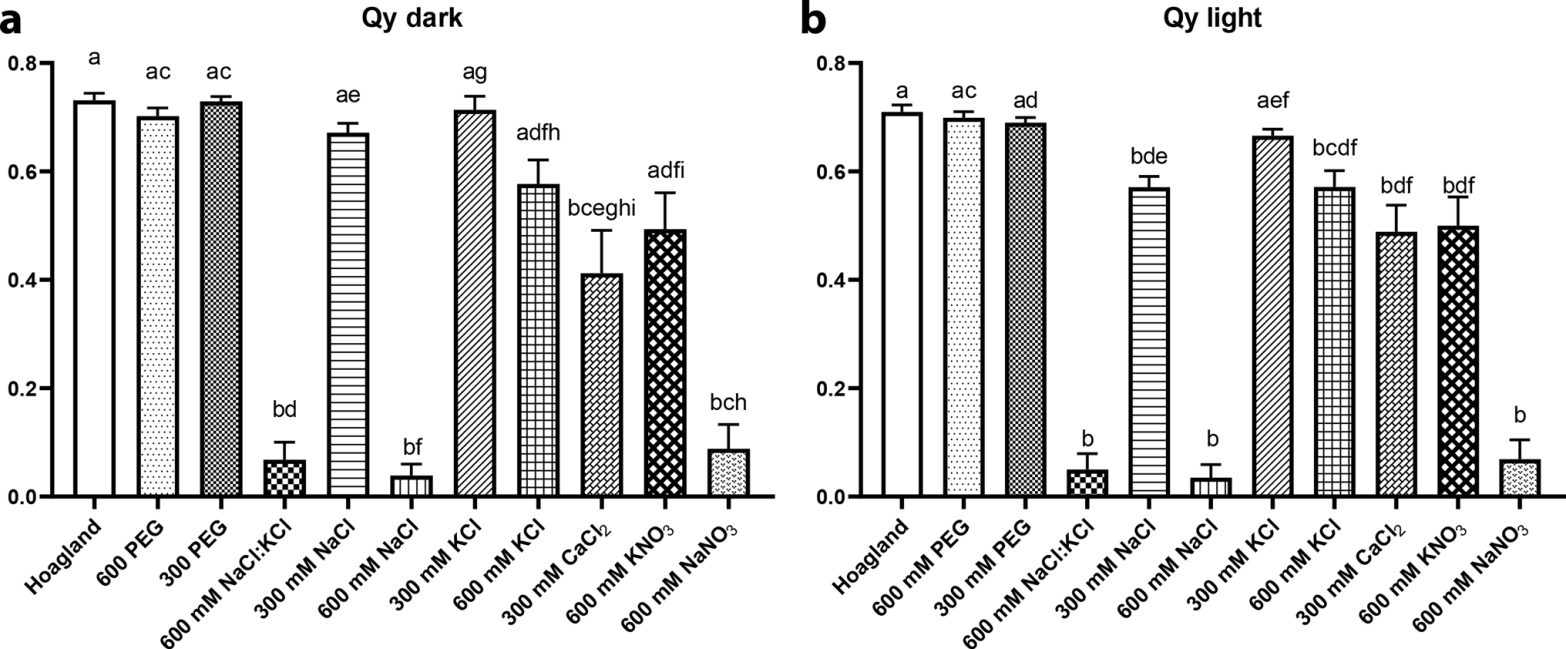
The maximal (Qy dark) and actual (Qy light) quantum efficiency of PS II of the wheat (*Triticum aestivum* L. cv. Mv Béres) leaf segments floated on various solutions for 1.5 h in the dark and then greened for 16 h on the same solution. Applied solutions: Hoagland, 600 PEG, 300 PEG, 600 mM NaCl:KCl (1:1), 300 mM NaCl, 600 mM NaCl, 300 mM KCl, 600 mM KCl, 300 mM CaCl_2_, 600 mM KNO_3_, 600 mM NaNO_3_. Plants were dark-adapted for 20 min before the Qy dark data were recorded. Error bars represent standard error. Different letters indicate statistically significant differences between the samples according to Kruskal–Wallis non-parametric ANOVA followed by Dunn’s multiple comparisons test (*P* < 0.05) (Qy dark, *n* = 10–20; Qy light, *n* = 10–33)

The Q_y_ dark parameter characterises the maximal quantum efficiency of the PSII after 20 min of dark adaptation, whilst Q_y_ light expresses the actual quantum efficiency of PSII as measured in the light-adapted state. In the case of 600 mM NaCl:KCl, 600 mM NaCl, and 600 mM NaNO_3_ samples, in which the Chl–protein complexes of the photosynthetic apparatus were not formed (Fig. 1d), the observed values are close to zero (Table S2, Fig. 5).

In the samples treated with 600 PEG, 300 PEG, 300 mM NaCl, and 300 mM KCl, we observed similar values than in the control samples greened on Hoagland solution. In the case of the samples treated with 600 mM KNO_3_ or 600 mM KCl, as well as 300 mM CaCl_2_ solutions, the parameters characterising the activity of PSII showed intermediate values (Table S2, Fig. 5). We did not observe large differences between the Qy light and Qy dark values measured for the same samples, which could be explained by the fact that due to the light-sensitivity and potential photo-oxidation of the developing photosynthetic apparatus and its Chl contents (Hideg et al. 2010), only relatively low-light values were used for light adaptation of the samples and also for the measurements.

### Plastid ultrastructure

**Fig. 6:**
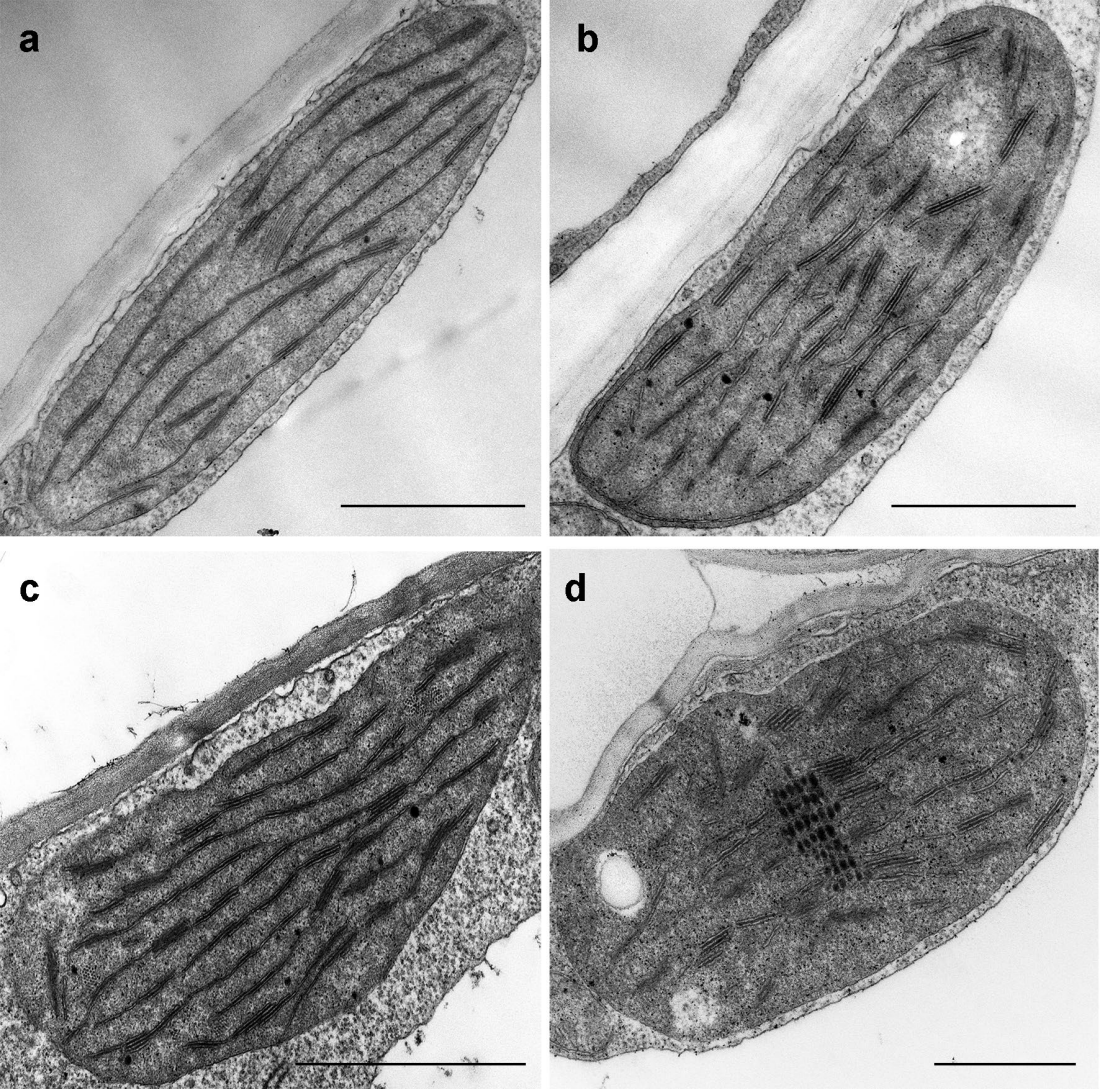
Typical plastid ultrastructure after 1.5 h dark pretreatment followed by 16 h greening of wheat (*Triticum aestivum* L. cv. Mv. Béres) leaf segments floating on different solutions: Hoagland (**a**), 600 PEG (**b**), 300 PEG (**c**), 300 mM KCl (**d**). Bar = 1 μm

As a result of light treatment in the control (Hoagland) and PEG-treated leaf segments (both in 300 PEG and 600 PEG), etioplast-to-chloroplast transformation proceeded normally (Fig. 5a–c) similarly to literature data (Abdelkader et al. 2007a). Elongated young chloroplasts with grana containing 2–3 thylakoid layers on average were observed in these leaves. Samples treated with PEG had a slightly more electron-dense stroma than the samples greened on Hoagland. Similar chloroplast ultrastructure was observed in the samples floated on 300 mM KCl during greening, with sometimes clusters of plastoglobuli indicating the previous presence of the PLB (Fig. 6d).

**Fig. 7:**
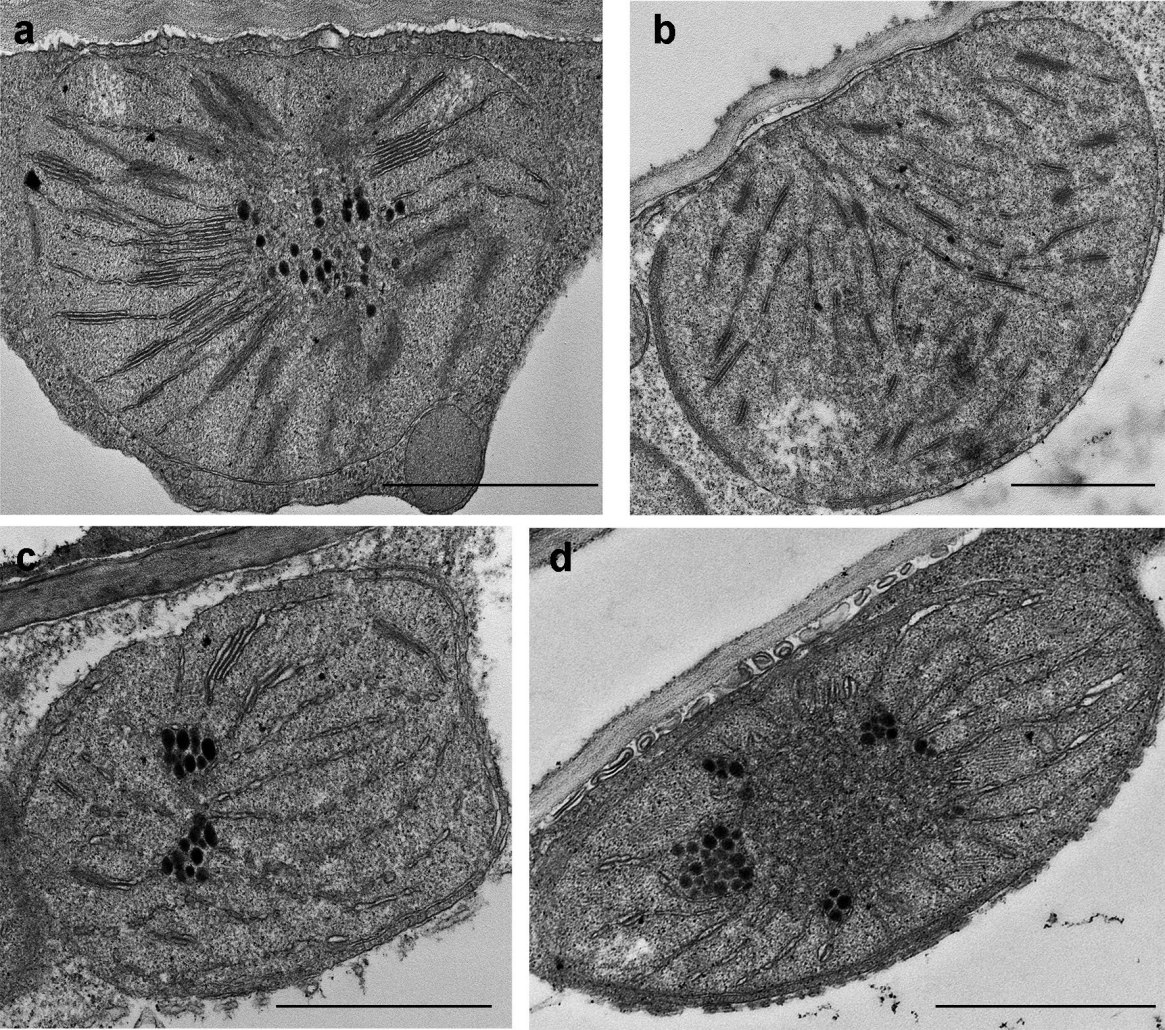
Typical plastid ultrastructure after 1.5 h dark pretreatment followed by 16 h greening of wheat (*Triticum aestivum* L. Cv. Mv. Béres) leaf segments floating on different solutions: 300 mM NaCl (**a**), 300 mM CaCl_2_ (**b**), 600 mM KNO_3_ (**c**), 600 mM KCl (**d**). Bar = 1 μm

In the case of samples which showed partial greening (Table 1, Fig. 7), such as 300 mM NaCl, 300 mM CaCl_2_, 600 mM KNO_3_, or 600 mM KCl solutions, the etioplast-to-chloroplast transformation also proceeded, although was not completed within 16 h. Remnants of PLBs interconnected with grana and stroma thylakoids of the developing so-called etio-chloroplasts were present in these samples with the exception of CaCl_2_ (Fig. 7). It is important to note, that granum development proceeded the most in the samples treated with 300 mM NaCl (Fig. 7a), in line with the fluorescence emission spectra (Fig. 1), and the presence of the Chl–protein complexes of the photosynthetic apparatus (Figs. 2, 3). In the samples greened in the presence of 300 mM CaCl_2_, no PLBs were observed (Fig. 7b) and their spectral characteristics resembled those of green leaves (Fig. 1b), but granum development was somewhat hindered, especially when compared with samples treated with 300 mM NaCl. The PLBs were retained in samples treated with 600 mM KNO_3_ and 600 mM KCl (Fig. 7c-d), but in accordance with their low Chl content (Table 1), no granum formation was observed in these samples.

**Fig. 8:**
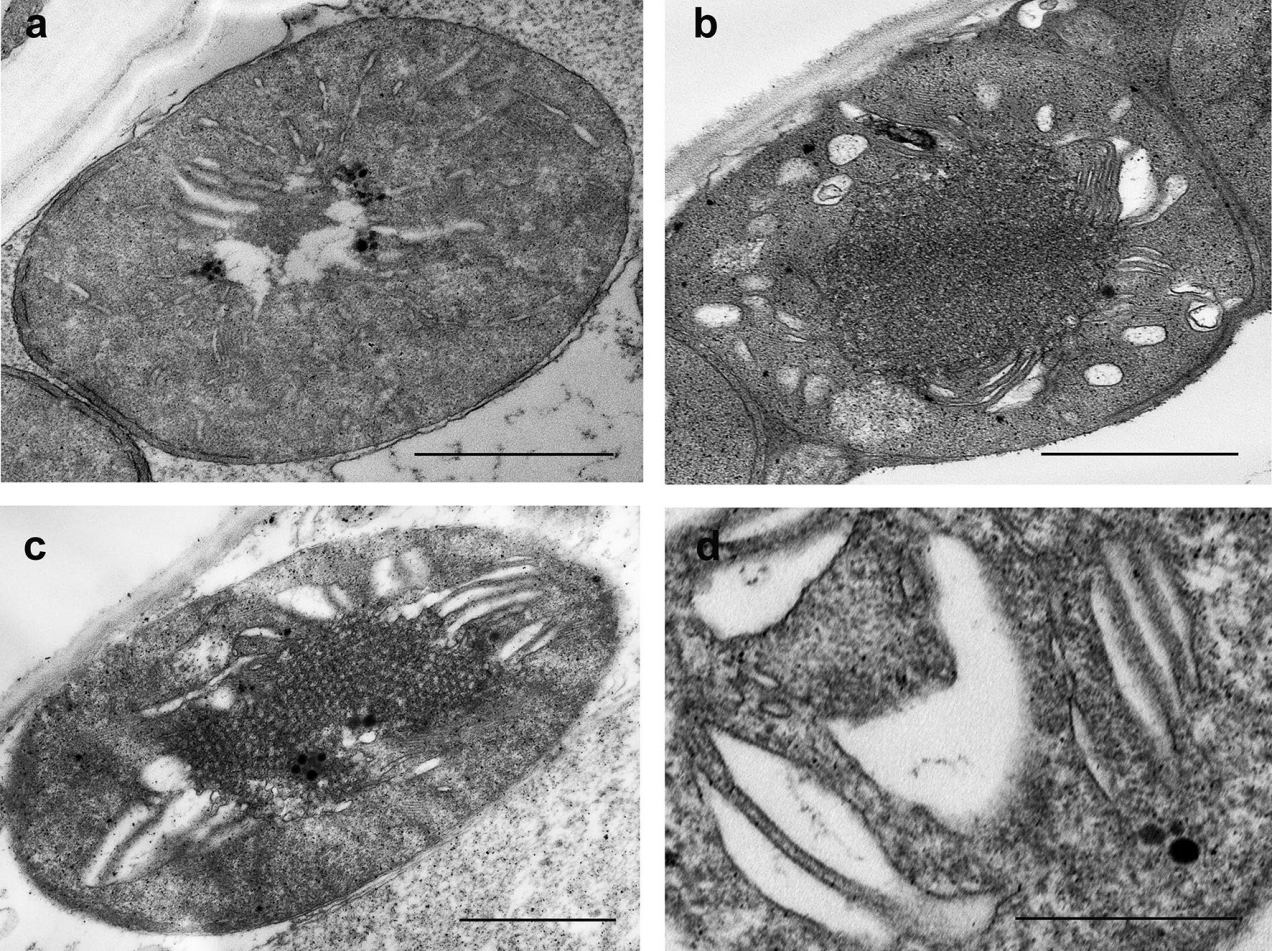
Typical plastid ultrastructure after 1.5 h dark pretreatment followed by 16 h greening of wheat (*Triticum aestivum* L. Cv. Mv. Béres) leaf segments floating on different solutions: 600 mM NaCl:KCl (1:1) (**a**), 600 mM NaCl (**b**), 600 mM NaNO_3_ (**c−d**). **d** The characteristic swelling observed in all samples. Bar = 1 μm **(a−c**) and 0.5 μm (**d**)

In samples treated with 600 mM NaCl:KCl, 600 mM NaCl or 600 mM NaNO_3_, Chl synthesis (Table 1) and the formation of the Chl–protein complexes (Fig. 1) were strongly hindered, the transition of the etioplasts to chloroplasts was also fully inhibited (Fig. 8a–d). In these cases, we usually observed PLB remnants inside the plastids, extensive and typical swelling of the (pro)thylakoid lumen resembling that observed in leaf segments treated with 600 mM NaCl:KCl for 4 h in the dark (Abdelkader et al. 2007a).

## Discussion

Wheat is one of the most important crops cultivated worldwide. It is therefore of crucial importance to understand how soil salinity affects its growth. In this respect, most studies compare seedling growth, photosynthetic parameters or grain yields and quality of the various wheat cultivars of germinating seedlings or plants treated by salt stress via the roots (Munns et al. 2012; Janda et al. 2016; Darko et al. 2019). In the present work, we have investigated a salt-sensitive Hungarian wheat cultivar (Janda et al. 2016; Darko et al. 2019), but we were interested about how and whether osmotic stress and various ions affect the greening, e.g. the etioplast-to-chloroplast transformation in wheat leaves in case the latter are directly exposed to soil salinity. This question may be environmentally relevant as wheat seeds are sown deep in the soil, where they are etiolated in the first few days of their development, and only start greening when they reach the soil surface.

Earlier data indicated that the greening of wheat leaf segments was inhibited and the intrathylakoid space of the prothylakoids of etioplasts showed unusual dilatation and swelling when the leaves were pretreated for 1.5 h in the dark by floating on 600 mM NaCl:KCl (1:1), and were then illuminated in the same solution for 8 h with relatively low-light intensities (50 μmol photons m^−2^ s^−1^) (Abdelkader et al. 2007a). However, it was unclear (1) whether the observed changes were associated with the high osmolarity and thus the osmotic stress caused by the above salt solution, or by its ionic effects, e.g. direct toxicity of the added ions (K^+^, Na^+^, or Cl^−^), and (2) in the latter case, by which ion(s). Therefore, we used non-ionic iso-osmotic solutions of PEG to decipher the osmotic component of the applied salt stress treatment, and also investigated the effect of various salt solutions on the Chl accumulation and greening of etiolated leaf segments greened for 16 h under the same conditions. For practical reasons, in this work, we used a Hungarian salt-sensitive wheat cultivar (Mv Béres) instead of the salt-sensitive Giza 168 wheat cultivar described earlier (Abdelkader et al. 2007a).

Treatments with isosmotic 300 or 600 PEG induced only mild and moderate effects on thylakoid and plastid development. Chl accumulation was reduced only moderately (Table 1), the photosynthetic activity (OJIP curves and Qy parameters, Figs. 4, 5) and the etio-chloroplast structure were similar to the Hoagland treated samples (Fig. 6). However, the greening process was a little slowed down in 600 PEG samples containing less PSII-s and LHCII and more CP43-less PSII compared to the control (Figs. 2, 3, S2). The high amount of CP43-less PSII may either represent a more intense PSII biogenesis (Rokka et al. 2005) or higher repair processes of PSII (Aro et al. 2005) in these samples. A similar tendency was observed with the photosynthetic activity measurements (Figs. 4, 5), which indicated that the osmotic component of the applied salt stress as well as low-concentration ion treatments have no significant effect on the physiological parameters of the leaves (Darko et al. 2019) even though the protein composition of the photosynthetic apparatus was slightly altered by the above treatments (Figs. 2, 3). In spite of the above-mentioned differences in thylakoid composition, these leaves showed 77 K fluorescence emission spectra similar to those samples treated on Hoagland (Fig. 1). This may be connected with the fact that the stage of thylakoid development has gone to the second phase: after the early integrated core complexes assembled with their antennae in the first phase, total complexes were built in the thylakoids in the second phase of greening (Kanervo et al. 2008; Rudowska et al. 2012). Also, in samples treated with 300 or 600 PEG, etioplast-to-chloroplast transformation proceeded similarly to the control (Fig. 6). On the basis of these results, we may conclude that the osmotic component does not seem to be very harmful to etioplasts and the greening process of wheat seedlings.

In contrast to PEG, the salt stress treatment, i.e. the synergetic effects of osmotic and ionic stress, seem to have a detrimental effect even when salts are applied in relatively low concentration. Though low concentration treatment with two essential nutrient ions (300 mM KCl, K^+^ being a macronutrient and Cl^−^ a micronutrient) had almost no effect on the observed processes, 300 mM NaCl, 300 mM CaCl_2_, 600 mM KCl, and 600 mM KNO_3_ treatments slowed down the greening process to different extent when compared with the control (Hoagland) samples. However, when high-concentration sodium containing salt solutions (600 mM NaCl, 600 mM NaNO_3_, 600 mM NaCl:KCl 1:1) were applied, the etioplast-to-chloroplast transformation was fully inhibited.

In leaf segments treated with 300 mM KCl or 300 mM NaCl, Chl synthesis was mildly or moderately inhibited (Chl *a/b* hardly changed) (Table 1), small grana and stroma thylakoid lamellae developed (Figs. 6d, 7a). Their 77 K fluorescence emission spectra showed the presence of the Chl–protein complexes of green leaves (Fig. 1), and their photosynthetic activity was similar to control samples greened in Hoagland solution (Figs. 4, 5). These leaf segments contained somewhat reduced amounts of PSI, PSII, and LHCII (Figs. 2, 3, S2) similarly to literature data of salt-treated green or developing plants (Srivastava and Sharma 2021; Zhu et al. 2021; Dhokne et al. 2022). The inhibition of PSII repair may contribute to the PSII decline as it was shown in salt-stressed cyanobacteria (Allakhaverdiev et al. 2002). It is also noteworthy that the ratio of the short- and long-wavelength fluorescence band was affected by 300 mM NaCl treatments (Table S1), whilst the ratio of PSII to PSI did not change significantly. This might indicate that salt treatment abolishes energy spillover from PSII to PSI as reported in the literature (Grieco et al. 2015; Kim et al. 2023).

Samples treated with 300 mM CaCl_2_, 600 mM KCl and 600 mM KNO_3_ represent an intermediate stage of thylakoid development. In the latter samples, similarly to those treated with 300 mM NaCl, PLBs persist even after 16 h of greening (Fig. 7). These samples showed low Chl content and a Chl *a/b* ratio higher than the control (Table 1) indicating some differences in the accumulation of Chl *a* and Chl *b* in these samples*.* Though salt stress is assumed to restrain individual steps of porphyrin formation, thereby inhibiting the accumulation of Chl (Abdelkader et al. 2007b), the lower accumulation of Chl *b* is probably due to the fact that the accumulation of the Chl *a*-containing core complexes precedes those of the Chl *a* and *b* containing antennae (Kanervo et al. 2008; Rudowska et al. 2012). Similarly, studies dealing with chlorina mutants of wheat confirmed that lowered Chl synthesis leads to selective decrease in Chl *b* contents (Falbel et al. 1996; Zivcak et al. 2019).

77 K fluorescence emission properties (Fig. 1), photosynthetic activity (Fig. 4b), and plastid structure (Fig. 7) of 600 mM KCl, and 600 mM KNO_3_, and 300 mM CaCl_2_ treatments refer to some thylakoid development, but the thylakoid complex composition of these samples was not investigated due to their low Chl content (Table 1). Their intermediate fluorescence emission spectra (Fig. 1) were similar to those observed in primary leaves of bean at the end of the first light period of their development (Schoefs and Franck 2008), and in the leaf primordia of various buds (Solymosi and Böddi 2006; Solymosi et al. 2012). As the greening progresses in leaves, a decrease in the ratio of the short- to the long-wavelength Chl bands is observed (Hák et al. 1990). In addition, in the spectra of these samples, a shoulder at 720 nm was evident (Fig. 1c), which may arise from the presence of PSI core complexes, in addition to the main fluorescence band at around 740 nm originating from PSI complexes with bound antennae (PSI-LHCI) (Mullet et al. 1980; Kalaji et al. 2017). This may indicate, that at this early phases of the differentiation of the photosynthetic machinery, a surplus of core complexes is present, which is not necessarily associated with LHCI.

Concerning photosynthetic activity, when compared with samples greened in Hoagland solution, the J–I phase of the OJIP curves of these intermediately greened samples is flatter, especially for 600 mM KCl and 300 mM CaCl_2_, which may mean that either the Q_A_ to Q_B_ electron transfer is less efficient, or electron flow to PSI and PSI acceptors is more efficient in these samples. In these samples, points J and P were reached later, the P680 system reached the closed state (P) in general later (Phi_Pav = t_Fm_ = time to reach the maximal fluorescence intensity) (Table S2). Similar observations were made under short-term salt treatment in Arabidopsis (Dukic et al. 2019), and may indicate important differences in the filling of the plastoquinone pool.

In samples with the lowest Chl concentrations, Chl biosynthesis was significantly inhibited as described earlier in a different wheat variety in case of 600 mM NaCl:KCl (1:1) treatment (Abdelkader et al. 2007a, b). The amount of the newly formed Chl was in the range of Pchlide and other Chl biosynthesis precursors present in the leaves. In the spectra of such leaves (Fig. 1d), the fluorescence emission maximum was at around 680 nm, which was similar to the band that appears after the phototransformation of Pchlide followed by the Shibata shift (Smeller et al. 2003). Alternatively, fluorescence emission at 680 nm may arise from free LHCII monomers and trimers which cannot transfer their energy to PSII (Kalaji et al. 2017). However, ATP synthase and Cyt *b/f* complexes were detected in the isolated thylakoids (Fig. S1) but Chl–protein complexes could not be isolated in distinguishable amounts by gel electrophoresis similarly to data in etioplasts or other samples at the beginning of greening (Mysliwa-Kurdziel et al. 1997; Kanervo et al. 2008). Furthermore, the photosynthetic activity (Qy data, Fig. 5, Table S2) was close to zero in these samples. Photosynthetic activity (of PSII) can be detected in etiolated wheat leaves within approx. 4 h after greening starts (Kanervo et al. 2008), but in our samples, the development of the Chl–protein complexes of the photosynthetic apparatus was probably very strongly inhibited by salt stress as observed in Abdelkader et al. (2007a). Therefore, although we cannot determine the exact origin of the fluorescence of the 680 nm band in these samples, our results indicate that it is rather associated with Chlide bound to LPOR than arising from LHCII. The data indicate that only the transformation of Pchlide to Chl could proceed in these plants, and no further development of the photosynthetic apparatus was observed. Thus, we may speculate that high concentrations of Na^+^ (and maybe Cl^−^ ions) stabilise the protein conformation of the LPOR enzyme (Hani et al. 2019) or the plastid inner membranes as shown for instance for grana in chloroplasts (Ünnep et al. 2014) or inhibit the release of the Chl(ide) bound to LPOR. Cryo-electron microscopic investigations have shown that the PLB structure is stabilised by the presence of LPOR bound to its outer surface in helically arranged oligomers (Floris and Kühlbrandt 2021). Therefore, the potential effect of NaCl on the hydrophilic–hydrophobic protein interactions, especially of the stroma-exposed regions of LPOR may contribute to the preservation of the PLB structure and the inhibition of the next steps of greening and the overall Chl synthesis (Fig. 8a, b).

Although the impact of 300 mM NaCl or KCl treatment was surprisingly mild, indicating that wheat leaves indeed have a relatively strong salt tolerance under our experimental conditions, applying the salts together (600 mM NaCl:KCl 1:1) resulted in similarly detrimental effects, i.e. swelling of the intrathylakoid space, as after 600 mM Na^+^ treatment. This may refer to the fact that the effect of salt stress should be rather examined quantitatively (e.g. in the context of the applied concentrations), than in the qualitative context (comparing the effects of various ions). Therefore, we cannot exclude that at higher osmolarity values or at higher applied salt (e.g. KCl, KNO_3_ or CaCl_2_) concentrations, similar alterations would appear.

The retardation of PLB transformation and thylakoid development under salt stress may be related to the impact of salinity on the biosynthesis of the pigment, protein and/or lipid components of the latter, which are strongly interrelated in thylakoid biogenesis (Kanervo et al. 2008; Solymosi and Aronsson 2013; Solymosi and Mysliwa-Kurdziel 2021). In the case of CaCl_2_ and the K^+^ treatments (300 mM KCl, 600 mM KCl and KNO_3_), probably the membrane protecting and Na^+^ homeostasis regulating effects of Ca^2+^ (Lachmann and Kesselmeier 1989; Marschner 1995; Yang et al. 2019) and the potential restoration of the Na^+^-induced K^+^ deficiency of chloroplasts by K^+^ (Slabu et al. 2009; Che et al. 2023) may be responsible for the milder salt stress effects, respectively. Literature data reported that CaCl_2_ stabilises PLB structure during greening (Lachmann and Kesselmeier 1989). However, those experiments were performed in vitro on isolated oat PLBs and etioplasts, and the used concentrations were much lower than in our case (up to 20 or 50 mM). PLB and etioplast isolation is performed in various buffered and ionic solutions; therefore, results obtained in such isolated, in vitro systems might not be comparable with the used experimental setup in this work.

Our data indicated that the observed strong inhibition of Chl accumulation and etioplast-to-chloroplast transformation in leaf samples treated with 600 mM NaCl, NaNO_3_ and NaCl:KCl (1:1) solutions were not related to the osmotic component of the applied salt stress, because in the isosmotic 600 PEG solution, chloroplast development occurred similarly to the control (Hoagland) sample (Fig. 5a and b). This led us to the conclusion that the swelling is induced by the specific ionic component of the applied salt solutions. As our original observation was carried out in a complex solution containing 300 mM NaCl and 300 mM KCl (Abdelkader et al. 2007a), we wanted to elucidate whether Na^+^, K^+^, or Cl^−^ or a combination of these was responsible for the observed ultrastructural changes. Our data showed that swelling occurred in samples floated on 600 mM NaCl or NaNO_3_ (Fig. 8b and c), but was absent in samples containing 600 mM KCl or KNO_3_, 300 mM CaCl_2_ (Fig. 7b–d), and 300 mM NaCl (Fig. 7a) or KCl (Fig. 6d) suggesting that the peculiar swelling of the (pro)thylakoid lumen is observed in samples containing Na^+^ as cation at relatively high salt concentrations (600 mM) or in the presence of other ions (600 mM NaCl:KCl, 1:1), in the case of which the synergetic effect of the various ions should be also considered. Interestingly, a similar phenomenon was observed in the inner mitochondrial membrane, in which Na^+^/H^+^ antiporter activity was suggested to be responsible for the selective swelling caused by Na^+^, but not by K^+^ (Bernardi 1999).

Numerous ion channels, transporters and ion pumps located in the plastid envelope and in the thylakoid membranes are responsible for the ion homeostasis of these organelles (Szabó and Spetea 2017). In the context of this work, cations may be transported across the thylakoids by several components, from which few are already characterised: a moderately voltage-dependent non-selective cation channel (Pottosin and Schönknecht 1996), a K^+^/H^+^ exchanger (KEA3) (Kunz et al. 2014; Dukic et al. 2019) or a Na^+^/H^+^ exchanger (NHD1) (Tomizioli et al. 2014). In addition, voltage-gated chloride channels (VCCN1 and VCCN2) and a member of the Cl^−^ channel CLC family (CLCe) may be responsible for chloride transport (Herdean et al. 2016a, b). The above ion transport components play an important role in the maintenance of chloroplast structure and function under natural conditions, including the maintenance of the proton motive force under fluctuating light conditions (Kunz et al. 2014; Herdean et al. 2016b, a; Dukic et al. 2019). It might be possible that accumulation of Cl^−^ in the lumen is responsible for the swelling of the intrathylakoid space, whilst the influx of Na^+^ or K^+^ from the lumen to the stroma causes shrinkage (Pottosin and Shabala 2016). However, further investigations would be necessary to decipher the presence and the activity of these ion transport components in the prothylakoids of etiolated wheat leaf etioplasts, and their potential involvement in the swelling induced by excess Na^+^ ions.

## Conclusions

As the 300 and 600 isosmotic PEG treatments only had slight and moderate impacts on the development of the photosynthetic apparatus and etioplast–chloroplast transition, the major effects of salt stress observed during the early stage of greening (swelling of the prothylakoid lumen, inhibition of Chl accumulation, of etioplast-to-chloroplast transformation, and of the development of the photosynthetic apparatus) can be attributed to specific ionic interactions depending on the used cations, their concentration, and the accompanying anions. High concentration of neither K^+^ (600 mM) nor Ca^2+^ (300 mM), but Na^+^ (600 mM) had the most detrimental effect on the greening process and on etioplast structure. Also, under our experimental conditions, the typical swelling of the (pro)thylakoid lumen was strongly associated with the presence of high concentrations of Na^+^ ions, possibly indicating altered ion transport and homeostasis of these membranes. Regarding the anions, NO_3_^−^ had a more favourable effect on thylakoid development than Cl^−^.

In a broader sense, our data may indicate that in comparison with the other ions used in this work, KCl or CaCl_2_, especially when applied in low concentrations, might represent environmentally friendly alternatives for salting roads. They do not affect or only mildly influence, respectively, the structure and the function of etioplasts of seedlings germinating in the soil, as well as their greening process once they reached the soil surface.

### Supplementary Information

Below is the link to the electronic supplementary material.

Supplementary file1 (DOCX 776 kb)

## Data Availability

The datasets generated during and/or analysed during the current study are available from the corresponding author on reasonable request.

## Abbreviations

BN PAGE: Blue Native polyacrylamide gel electrophoresis
Chlide: Chlorophyllide
Cyt: Cytochrome
LPOR: NADPH:protochlorophyllide oxidoreductase
LHC: Light-harvesting complex
Pchlide: Protochlorophyllide
PLB: Prolamellar body
