# Supplementary material for: Ionic, not the osmotic component, is responsible for the salinity-induced inhibition of greening in etiolated wheat (Triticum aestivum L. cv. Mv Béres) leaves: a comparative study
Source: Planta. 2023 Oct 20;258(5):102. doi: 10.1007/s00425-023-04255-4 (PMC10589150; doi:10.1007/s00425-023-04255-4)
Supplement: Supplementary file 1 — Supplementary file1 (DOCX 776 kb) [file 425_2023_4255_MOESM1_ESM.docx]

**Table S1**. Ratio of the fluorescence emission intensities of the short wavelength and long wavelength maxima of the spectra (indicated as F~680 and F~740, as their values varied between 676-697 and 720-745 nm wavelengths). Mean values with standard errors are provided. Different letters indicate statistically significant differences between the samples according to Kruskal-Wallis non-parametric ANOVA followed by Dunn’s multiple comparisons test (*P* < 0.05) (*n*=11-44).

|  | **Ratio of F~680/F~740** |
| --- | --- |
| **Hoagland** | **0.20 ± 0.01^ab^** |
| **300 PEG** | **0.20±0.01^a^** |
| **600 PEG** | **0.17±0.01^a^** |
| **300 mM KCl** | **0.24±0.01^ab^** |
| **300 mM NaCl** | **0.52±0.06^bc^** |
| **300 mM CaCl_2_** | **1.26±0.21^bc^** |
| **600 mM KNO_3_** | **2.22±0.32^ce^** |
| **600 mM KCl** | **2.77±0.38^cf^** |
| **600 mM NaNO_3_** | **4.82±0.22^df^** |
| **600 mM NaCl** | **4.93±0.26^def^** |
| **600 mM NaCl:KCl (1:1)** | **5.01±0.23^def^** |


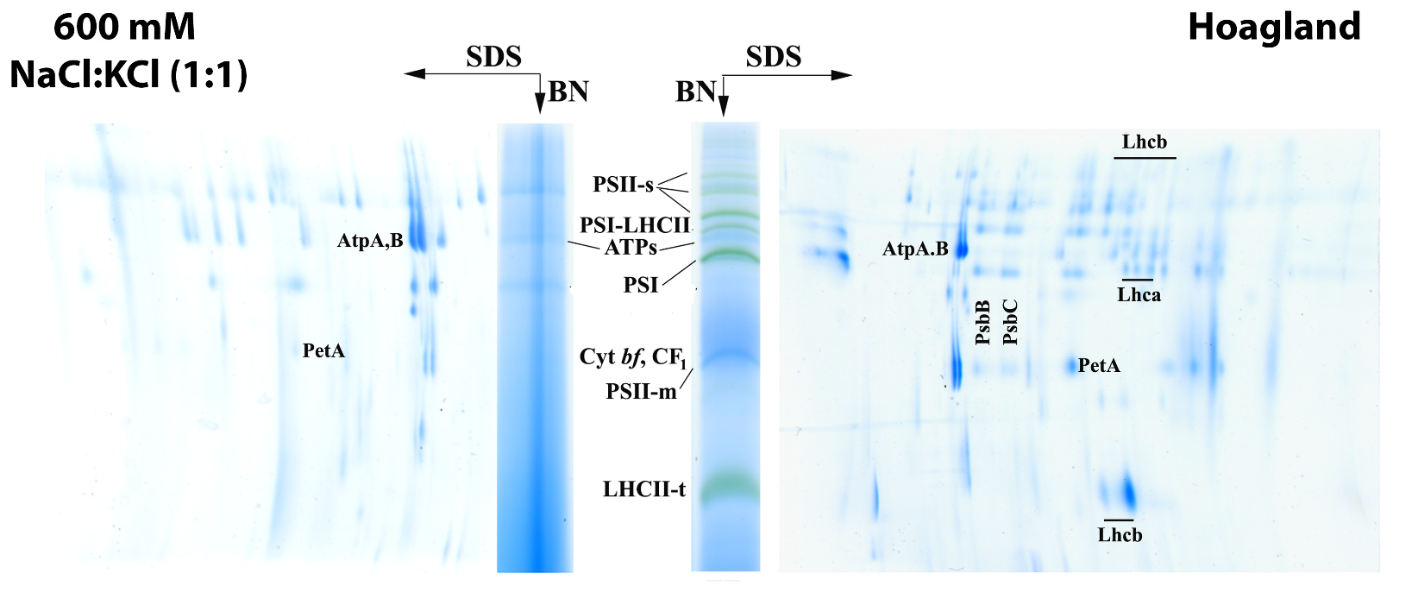
**Fig. S1**. Complexes present in thylakoids isolated from wheat (*Triticum aestivum* L. cv. Mv Béres) leaf segments floated on various solutions for 1.5 h in the dark and then greened for 16 h on the same solution. Applied solutions: Hoagland and 600 mM NaCl:KCl (1:1). Thylakoids (Hoagland – 250 µg Chl ml^-1^ and 600 mM NaCl:KCl (1:1) of similar protein content) were solubilized using 2% n-dodecyl-*β*-D-maltoside plus 1% digitonin and separated in 4.5-12% gel gradient followed by SDS PAGE. PS – photosystem, LHC – light-harvesting complex, s – supercomplex, t – trimer, Cyt *bf* – cytochrome *b_6_f* complex dimer, ATPs – ATP synthase.

*
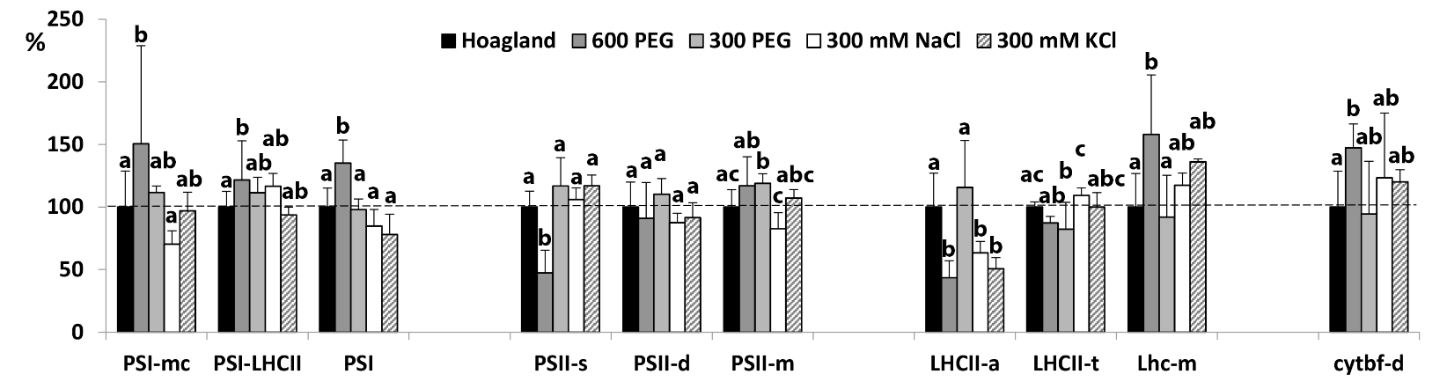
*

**Fig. S2.** Changes in the amounts of thylakoid complexes expressed as the percentage of the control (Hoagland) values in the wheat (*Triticum aestivum* L. cv. Mv Béres) leaf segments floated on various solutions for 1.5 h in the dark and then greened for 16 h on the same solution. Applied solutions: Hoagland, 600 PEG, 300 PEG, 300 mM NaCl, 300 mM KCl. Thylakoids (250 µg Chl ml^-1^, µg protein µg^-1^ Chl ~ 10) were solubilized using 2% n-dodecyl-*β*-D-maltoside plus 1% digitonin and separated in 4.5-12% gel gradient. PS – photosystem, LHC, Lhc – light-harvesting complex, LHCII-a – LHCII-assembly: CP29-CP24-LHCII trimer, mc – megacomplex, s – supercomplex, t – trimer, d – dimer, m – monomer, cytbf – cytochrome *b_6_f* complex. The lanes were normalized to the same sum pixel number. Error bars represent SD values. Different letters indicate statistically significant differences between the samples according to Tukey’s multiple comparisons test (*P* < 0.05) (*n*=3-20).

**Table S2.** Overview and characteristics of the chlorophyll fluorescence parameters (Vi, Vj, and t_Fm_) derived from the fast chlorophyll fluorescence kinetics (OJIP) and the values of Qy dark and Qy light. Mean values with standard errors are provided. Different letters indicate statistically significant differences between the samples according to Kruskal-Wallis non-parametric ANOVA followed by Dunn’s multiple comparisons test (*P* < 0.05) (OJIP, *n*=9-18; Qy dark, *n* =10–20; Qy light, *n* =7-17).

|  | **Vi** | **Vj** | **Phi_Pav** | **Qy dark** | **Qy light** |
| --- | --- | --- | --- | --- | --- |
| **Hoagland** | 0.74±0.01^ab^ | 0.58±0.01^abc^ | 906.9±3.92^a^ | 0.73±0.01^a^ | 0.73±0.01^a^ |
| **300 mM PEG** | 0.71±0.01^abc^ | 0.57±0.01^abc^ | 911.6±2.70^ab^ | 0.73±0.01^ac^ | 0.69±0.01^ad^ |
| **600 mM PEG** | 0.72±0.01^ab^ | 0.58±0.01^abc^ | 916.4±3.45^ab^ | 0.70±0.01^ac^ | 0.70±0.01^ac^ |
| **300 mM KCl** | 0.76±0.00^a^ | 0.57±0.01^ac^ | 916.1±2.57^ab^ | 0.71±0.02^ag^ | 0.67±0.01^aef^ |
| **300 mM NaCl** | 0.68±0.05^ab^ | 0.61±0.01^b^ | 922.2±2.62^ab^ | 0.67±0.02^ae^ | 0.57±0.02^bde^ |
| **300 mM CaCl_2_** | 0.64±0.02^bd^ | 0.62±0.01^bc^ | 931.9±5.68^b^ | 0.41±0.08^bceghi^ | 0.49±0.05^bdf^ |
| **600 mM KNO_3_** | 0.55±0.03^ad^ | 0.55±0.02^ab^ | 938.2±15.32^b^ | 0.49±0.07^adfi^ | 0.50±0.05^bdf^ |
| **600 mM KCl** | 0.71±0.01^cd^ | 0.65±0.03^a^ | 940.1±9,562^ab^ | 0.58±0.04^adfh^ | 0.57±0.03^bcdf^ |
| **600 mM NaNO_3_** |  |  | - | 0.09±0.04^bch^ | 0.07±0.04^b^ |
| **600 mM NaCl** |  |  | - | 0.04±0.02^bf^ | 0.04±0.02^b^ |
| **600 mM NaCl:KCl (1:1)** |  |  | - | 0.07±0.03^bd^ | 0.05±0.03^b^ |
